# Supplementary material for: DENA: training an authentic neural network model using Nanopore sequencing data of Arabidopsis transcripts for detection and quantification of N6-methyladenosine on RNA
Source: Genome Biol. 2022 Jan 17;23:25. doi: 10.1186/s13059-021-02598-3 (PMC8762864; doi:10.1186/s13059-021-02598-3)
Supplement: Supplementary file 1 — Additional file 1: Fig. S1. Nanopore direct RNA-Seq implementation and m6A detection with differr tool. Fig. S2. Training DENA. Fig. S3. Confirming the reliability of DENA in m6A quantification. Fig. S4. The correlation of modification rate between wild-type and m6A-deficient A.thaliana mutant. Table S1 Sequencing statistics of poly(A) selected RNAs in biological triplicates from Col0, mtb, and fip37-4 using direct RNA-Seq, respectively. Table S2 The performance of the DENA prediction model that was evaluated with metrics including accuracy, recall, precision, F1-score. Table S3 The comparison of m6A modification rates between DENA and other methods (containing xPore, Nanom6A, SCARLET, LEAD-m6A- seq and Deoxyribozyme-based Method) at the previously identified m6A sites in human. NT: Not detected; -: Not identified; Y: identified as m6A site. Table S4 DNA probes used in the SELECT assay. [file 13059_2021_2598_MOESM1_ESM.pdf]

Supplementary information

DENA: training an authentic neural network model using Nanopore sequencing data of Arabidopsis transcripts for detection and quantification of N6-methyladenosine on RNA

Hang Qin[1,4,#], Liang Ou[2,4,#], Jian Gao[3,4], Longxian Chen[1,4], Jiawei Wang[3,4,*], Pei Hao[2,4,*], Xuan Li[1,4,*]

[1]Key Laboratory of Synthetic Biology, CAS Center for Excellence in Molecular Plant Sciences, Institute of Plant Physiology and Ecology, Chinese Academy of Sciences, Shanghai, China

[2]Key Laboratory of Molecular Virology and Immunology, Institut Pasteur of Shanghai, Chinese Academy of Sciences, Shanghai, China.

[3]National Key Laboratory of Plant Molecular Genetics, CAS Center for Excellence in Molecular Plant Sciences, Institute of Plant Physiology and Ecology, Chinese Academic of Sciences, Shanghai, China

[4]University of Chinese Academy of Sciences, Beijing, China

[#]These authors contributed equally to this work

* Corresponding author:

Xuan Li: lixuan@cemps.ac.cn

Pei Hao: phao@ips.ac.cn

Jiawei Wang: jwwang@cemps.ac.cn

Contents

Supplementary Figures

Supplementary Tables

Supplementary Figures

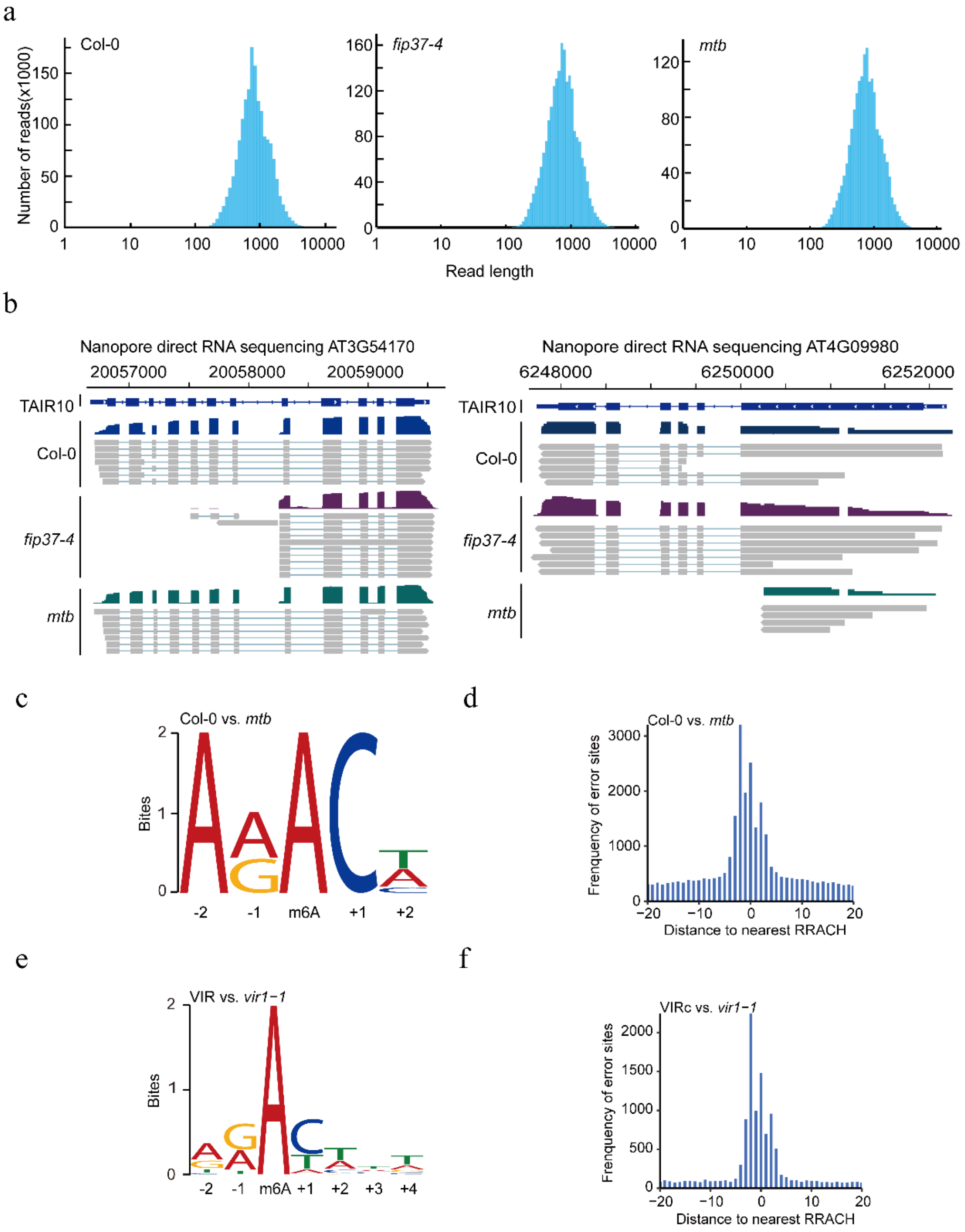

Fig. S1 Nanopore direct RNA-Seq implementation and m^{6}A detection with *differr* tool. (a) The distribution of direct RNA-Seq reads from Col-0, *fip37-4*, and *mtb*, respectively. (b) IGV shows the alignment of direct RNA-Seq reads for FIP37 (AT3G54170) and MTB (AT4G09980) gene in Col-0, *fip37-4*, and *mtb*, respectively. (c) and (e) are the consensus motifs in C*m* and V*v*, respectively. (d) and (f) are the distribution of distances between the differential sites and its nearest "RRACH" sequences in C*m* and V*v*, respectively.

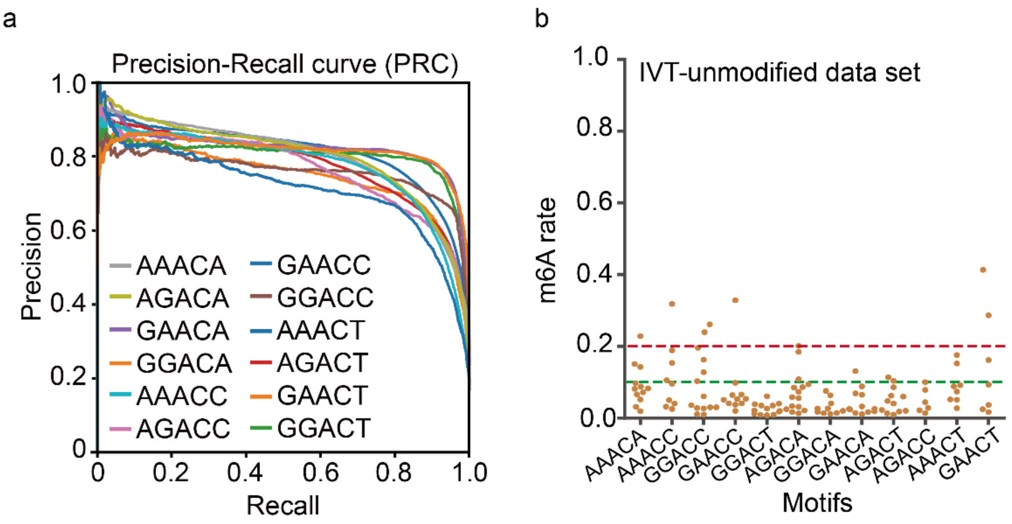

Fig. S2 Training DENA. (a) The Precision-Recall curve (PRC) of *DENA*. (b) The m^6A prediction for unmodified training data from *in vitro* synthetic RNAs using *DENA*. The green and red lines show the m^6A rate with 0.1 and 0.2, respectively.

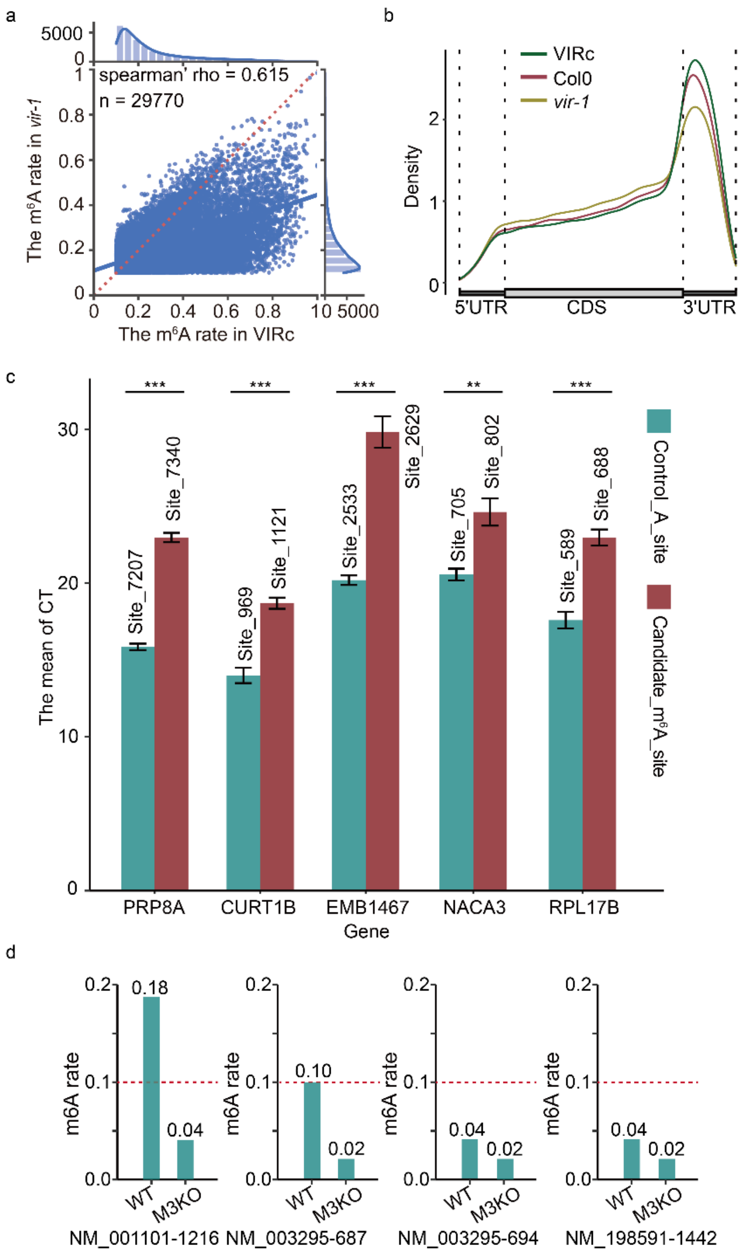

Fig. S3 Confirming the reliability of *DENA* in m^{6}A quantification. (a) Jointplot shows the correlation of m^{6}A rates from 29770 intersected sites between *vir-1* and VIRc. (b) The m^{6}A distribution on transcripts in Col-0, VIRc and *vir-1*, respectively. (c) The identification of five m^{6}A sites predicted by DENA using qPCR (real-time quantitative PCR). Green shows the “A” base of control. Red shows the DENA-predicted m^{6}A site. P values from t-test (two-tailed) are shown on top of the bar plots. *p < 0.05; **p < 0.01; ***p < 0.001. (d) Bar plot shows the m^{6}A rates identified by *DENA* at NM_001101-1216, NM_003295-687, NM_003295-694 and NM_198591-1442 sites in WT and M3KO cells, respectively.

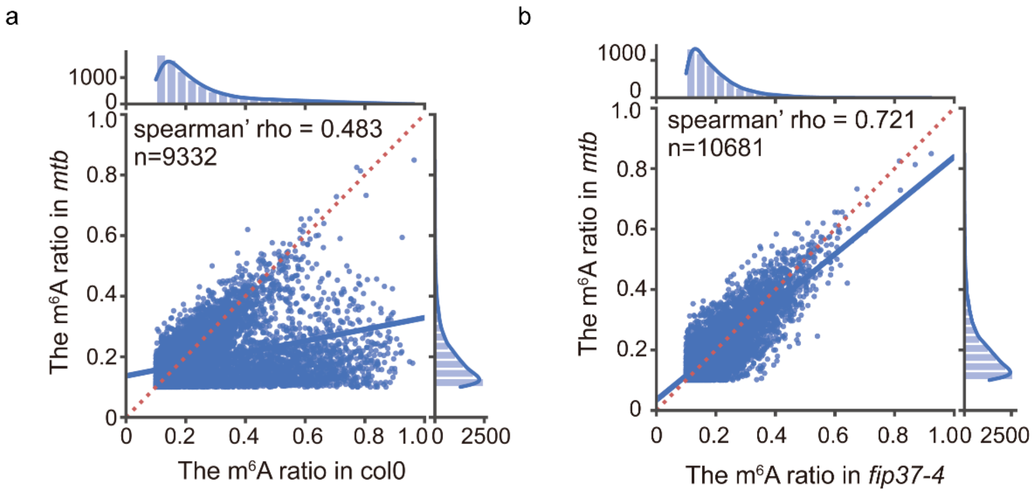

Fig. S4 The correlation of modification rate between wild-type and m^{6}A-deficient *A.thaliana* mutant. (a) Jointplot shows the correlation of m^{6}A rates at 9330 intersected sites between Col-0 and *mtb*. (b) Jointplot shows the correlation of m^{6}A rates at 29770 intersected sites between *fip37-4* and *mtb*.

Supplementary Tables

Table S1 Sequencing statistics of poly(A) selected RNAs in biological triplicates from Col0, *mtb*, and *fip37-4* using direct RNA-Seq, respectively.

		Col0			*fip37-4*			*mtb*		
		Col0_1	Col0_2	Col0_3	*fip37_1*	*fip37_2*	*fip37_3*	*mtb_1*	*mtb_2*	*mtb_3*
Raw data	Number of reads	355285	518005	1016370	621552	737486	1019317	380011	760082	758060
	Mean read length	1024.9	1044.1	740.1	891.5	906.9	682.7	916.5	913.3	731.2
	Mean read quality	7.8	7.8	7.7	8	7.9	7.6	7.9	8	7.7
	Median read length	859	874	638	774	785	577	787	786	616
	Median read quality	7.8	7.7	8	8	7.9	7.9	7.9	8	8
	Read length N50	1162	1191	895	1011	1024	883	1032	1034	928
	Total reads		1889660			2378355			1898153	
	Total aligned reads		1739015			2181217			1756890	
Quanlity Control (read-length >100bp and Q>7)	Number of reads	352600	513852	841035	617154	731679	810171	377050	754448	622851
	Mean read length:	1027.7	1046.8	806.9	895	910.7	775.9	920.1	917	812.6
	Mean read quality	7.8	7.8	8.1	8	7.9	8	7.9	8	8.1
	Median read length	861	876	698	776	787	663	789	788	692
	Median read quality	7.8	7.7	8.1	8	7.9	8	7.9	8	8.1
	Read length N50	1164	1193	918	1011	1024	918	1033	1035	962
	Total reads		1707487			2159004			1754349	
	Total aligned reads		1687722			2117431			1714419	

Table S2 The performance of the *DENA* prediction model that was evaluated with metrics including accuracy, recall, precision, F1-score.

Motifs	Accuracy	Precision	Recall	F1-score
AAACT	0.8738	0.7044	0.8782	0.7818
GAACT	0.8832	0.6204	0.9113	0.7382
GGACT	0.9182	0.7705	0.8978	0.8293
AGACT	0.8798	0.6100	0.9161	0.7324
GAACA	0.9264	0.7341	0.9510	0.8286
GGACC	0.9191	0.6912	0.9059	0.7841
AGACC	0.8563	0.6005	0.9078	0.7228
AAACA	0.8396	0.6908	0.8396	0.7579
AGACA	0.8706	0.6161	0.9047	0.7330
AAACC	0.8835	0.6574	0.8678	0.7481
GAACC	0.8931	0.6133	0.8588	0.7156
GGACA	0.9142	0.7657	0.9257	0.8381

Table S3 The comparison of m^{6}A modification rates between *DENA* and other methods (containing *xPore*, *Nanom6A*, *SCARLET*, *LEAD-m6A- seq* and *Deoxyribozyme*-based Method) at the previously identified m^{6}A sites in human. NT: Not detected; -: Not identified; Y: identified as m^{6}A site.

gene	genoLoci	isoforms	transLoci	motif	*xPore*		*Nanom6A*		*DENA*		*SCARLET*	*LEAD-m6A-seq*	*Deoxyribozyme*
					rate		rate		rate		rate	rate	
					**WT**	M3KO	**WT**	M3KO	**WT**	M3KO	**WT**	**WT**	**WT**
ACTB	5527743	NM_001101.5	1217	GGACT	**0.75**	0.18	**0.64**	0.13	**0.18**	0.04	**0.21**	**0.29**	**Y**
BSG	583239	NM_001322243.2	1340	GGACT	**NT**	NT	**0.67**	0.55	**0.29**	0.09	**0.06**	**0.55**	**-**
		NM_198589.3	1344	GGACT	**1**	0.24			**0.36**	0.09			
		NM_198591.4	1378	GGACT	**1**	0.19			**0.16**	0.02			
	583346	NM_001322243.2	1447	GAACT	**NT**	NT	**0.18**	0.17	**0.06**	0.02	**0.01**	**-**	**-**
		NM_198589.3	1451	GAACT	**NT**	NT			**0.04**	0.02			
		NM_198591.4	1485	GAACT	**NT**	NT			**0.06**	0.02			
TPT1	45337310	NM_001286273.2	874	GGACT	**NT**	NT	**0.42**	0.11	**0.11**	0.02	**0.15**	**-**	**-**
		NM_003295.4	709	GGACT	**NT**	NT			**0.10**	0.02			
	45337303	NM_001286273.2	881	AGACA	**NT**	NT	**0.12**	0.07	**0.08**	0.02	**0.04**	**-**	**-**
		NM_003295.4	716	AGACA	**NT**	NT			**0.04**	0.02			
	45337294	NM_001286273.2	890	GGACT	**NT**	NT	**0.79**	0.37	**0.17**	0.02	**0.01**	**-**	**-**
		NM_003295.4	725	GGACT	**0.82**	0.2			**0.21**	0.04			
MRPL20	1402080	NM_017971.4	529	GGACT	**0.69**	0	**0.77**	0.33	**0.56**	0.26	**-**	**-**	**Y**
YTHDF2	28743593	NM_016258.3	1504	AGACT	**1**	0.75	**0.96**	0.76	**0.51**	0.33	**-**	**0.40**	**-**
ACTG1	81511529	NM_001614.5	533	GGACT	**1**	0.23	**0.92**	0.34	**0.41**	0.12	**-**	**0.84**	**-**
		NM_001199954.3	652	GGACT	**0.95**	0.19			**0.37**	0.19			
SEC11A	84669674	NM_014300.4	949	AGACT	**NT**	NT	**0.22**	NT	**0.18**	0.03	**-**	**-**	**Y**
PARP1	226361173	NM_001618.4	3496	AGACT	**NT**	NT	**0.44**	0.47	**0.07**	0.05	**-**	**0.02**	**-**

Table S4 DNA probes used in the SELECT assay.

Name	Sequence
qPCR-A-F	5'agatcgagagtgagtcgtgtgaat
qPCR-m6A-F	5'agatcggaagagcgtagtgtatga
EMB1467_2533-Dp-A	5'caccgcaagctaaacccgagatacaattcacacgactcactctcgatct
EMB1467_2533-Up-A	5'cagcaggtgtgcaaattgcttataga
EMB1467_2629-Dp-m6A	5'cctaataagaacaatacaagatgccatcatacactacgctcttccgatct
EMB1467_2629-Up-m6A	5'gcttctacatgcaaatgtaaaaggg
PRP8A_7207-Dp-A	5'cgatgctcctcgtgatagaactcctttattcacacgactcactctcgatct
PRP8A_7207-Up-A	5'caggtgactccaggaaatgagtg
PRP8A_7340-Dp-m6A	5'ctatactgcaaaataaagctaaatcactcatacactacgctcttccgatct
PRP8A_7340-Up-m6A	5'gctcgccagtacaacatcttaca
CURT1B_969-Dp-A	5'ccttcattccaattcatgaatggccattcacacgactcactctcgatct
CURT1B_969-Up-A	5'cgcagctggtgttagattcttttga
CURT1B_1121-Dp-m6A	5'cttgaggaatttacaacactttgactcatacactacgctcttccgatct
CURT1B_1121-Up-m6A	5'gctgcgaccaaaacatctcatatat
NACA3_705-Dp-A	5'cggatttaggtggtaagctccattcacacgactcactctcgatct
NACA3_705-Up-A	5'agtagcagcatcaaagtaggaaaag
NACA3_802-Dp-m6A	5'ccaaacgtatagtatataatgtcatacactacgctcttccgatct
NACA3_802-Up-m6A	5'gcctcggcttctaaaatatgaaatg
RPL17B_589-Dp-A	5'cttggacttggcagccaatatattcacacgactcactctcgatct
RPL17B_589-Up-A	5'ttagagaaagaaagcttaagctgctga
RPL17B_688-Dp-m6A	5'cttacaaaacgattcgagctaaagtcatacactacgctcttccgatct
RPL17B_688-Up-m6A	5'cctcggcaagataaatctatccat
