# Supplementary material for: DENA: training an authentic neural network model using Nanopore sequencing data of Arabidopsis transcripts for detection and quantification of N6-methyladenosine on RNA
Source: Genome Biol. 2022 Jan 17;23:25. doi: 10.1186/s13059-021-02598-3 (PMC8762864; doi:10.1186/s13059-021-02598-3)
Supplement: Supplementary file 4 — Additional file 4. Review history. [file 13059_2021_2598_MOESM4_ESM.docx]

Review History

**First round of review**

**Reviewer 1**

**Were you able to assess all statistics in the manuscript, including the appropriateness of statistical tests used?**

Yes.

**Were you able to directly test the methods?**

Yes.

**Comments to author:**

In this submitted manuscript, the authors have developed a novel neural network called DENA for m6A quantification using the sequencing data of in vivo transcripts from Arabidopsis. The method for m6A quantification is interesting and important in the epigenetic field. Despite the results presented, there are several concerns which need further clarifications (see below):

1. Should add both Receiver operating characteristic curve (ROC) and Precision-Recall curve (PRC).

2. The performance of the DENA prediction model may also be evaluated with metrics including accuracy, recall, precision, F1-score, etc.

3. Suggest the authors to build the website of DENA，if possible.

4) Considering RNA m6A is also well-known in other plants, such as rice, tomato, maize, the authors may take into consideration of analyzing m6A sites in these species to broaden the impacts of DENA models.

5) Do you have any experiments, such as m6A IP-Qpcr, to confirm predicted sites of m6A by DENA?

**Reviewer 2**

**Were you able to assess all statistics in the manuscript, including the appropriateness of statistical tests used?**

No.

**Were you able to directly test the methods?**

No.

**Comments to author:**

This is an important paper addressing part of a problem in the epitranscriptomics field, the detection of modified nucleotides using direct RNA sequencing on the nanopore. The paper makes strong claims about the strength of the tool, but this is largely supported by their data.

DENA does appear to be a superior choice to many of the alternatives for calling modified sites in direct-RNA sequencing dataset. The software is accessible through a well documented github (though, some proof reading is needed there!) and appropriately easy to use through python.

The authors do a very important test of DENA against SCARLET, the current gold standard in site-specific m6A quantification, and the examples here do add credence to their claims that DENA outperforms other tools. Here however I think the manuscript needs more data to be shown, how does e.g xPore perform? What m6A rates do other transcripts show in scarlett and other software? I wouldn't expect perfect performance from DENA in all scenarios (this would be remarkable) but I think this is a really important addition to allow the field to choose the best tool as needed.

The manuscript is clear and well written, it is however necessary to expand on how the DENA software works, considering the average reader of GenomeBiology, rather than an expert reader in the machine learning field. The paper is a thorough exploration of how DENA performs, but only ~20 lines are dedicated to how it functions or how it was developed in the methods. This really does need to be expanded for the final publication.

A good paper and a nice piece of software. I think addressing the above will make it a valuable contribution to the field.

**Authors Response**

**Point-by-point responses to the reviewers’ comments:**

Reviewer #1:

In this submitted manuscript, the authors have developed a novel neural network called DENA for m6A quantification using the sequencing data of in vivo transcripts from Arabidopsis. The method for m6A quantification is interesting and important in the epigenetic field. Despite the results presented, there are several concerns which need further clarifications (see below):

*Response：Thank you for your constructive comments and suggestions on our work. In the revised manuscript, we have addressed all the comments and performed many additional data analyses suggested by the reviewer.*

(1) Should add both Receiver operating characteristic curve (ROC) and Precision-Recall curve (PRC).

*Response：We thank the reviewer for the comment that brought the issue to our attention.*

*We have computed and added the Receiver operating characteristic curve (ROC) in Figure 3b and Additional file 1: Supplementary Figure 2a. We have referred/discussed it in the revised manuscript (page 8, lines 165-169; red text; also refer to next question).*

(2) The performance of the DENA prediction model may also be evaluated with metrics including accuracy, recall, precision, F1-score, etc.

*Response: We appreciate the reviewer’s comment, and have supplemented additional results (for metrics including accuracy, recall, precision, F1-score, etc) that were used to evaluate the performance of the DENA prediction model (Additional file 1: Supplementary Table 2). We also added some discussion on the evaluation results in the revised manuscript (page 8, lines 165-169; red text).*

(3) Suggest the authors to build the website of DENA, if possible.

*Response: We appreciate the reviewer’s suggestion, which would facilitate users’ usage and broaden the impact of DENA. At present, we have submitted the codes of DENA to both GitHub (*[*https://github.com/weir12/DENA*](https://github.com/weir12/DENA)*) and zenodo (https://zenodo.org/record/5603381) with the detailed instruction, and users have open access to them and can easily perform analysis on their own data. We have added new texts on instructions how to access to and use them in the revised manuscript (page 28, lines 609-612; red text).*

*Per the reviewer’s comment, we have also investigated and drawn up a plan to build a website for DENA and extending its functionalities by including a database of RNA m6A modifications for model organisms like Arabidopsis, rice, tobacco, tomato, etc. The project is ongoing (requiring some extraordinary effort), and hopefully will be available to enhance DENA in the near future. Thank you for the valuable suggestion!*

(4) Considering RNA m6A is also well-known in other plants, such as rice, tomato, maize, the authors may take into consideration of analyzing m6A sites in these species to broaden the impacts of DENA models.

*Response: We thank the reviewer for the suggestion.*

*Per the comment, we searched many public databases, like NCBI and ENA, but found few nanopore direct RNA sequencing data available, besides the number of datasets of nanopore direct RNA sequencing we already obtained in our study and some data we generated ourselves.*

*We further investigated and found currently it would be some undertaking to perform nanopore direct RNA sequencing on new plant species. To broaden the impact of DENA in study of RNA m6A identification in other plant species, we are coordinating with our collaborators working on direct RNA sequencing of materials from rice, tomato, maize, etc. This is also part of the effort to extend its functionalities by including a database of RNA m6A modifications for model organisms we discussed in the previous question. Again, thank you for the valuable suggestion!*

(5) Do you have any experiments, such as m6A IP-Qpcr, to confirm predicted sites of m6A by DENA?

*Response: We appreciate the reviewer’s comment.*

*We had no experimental validation in the previously submitted version to confirm predicated sites of m6A by DENA. During revision of the manuscript, we made effort to adopt the experimental method, named SELECT [1], which can detect m6A modification at single-base resolution using DNA polymerase and DNA ligase combining with qPCR. On the selected sites of m6A modification that were detected using DENA, the qPCR data confirmed the results of m6A modification, supporting the reliability of DENA in prediction of m6A modification. The new experimental data were added to “Figure 4h, 4i” and “Additional file 1: Supplementary Figure 3c”, and were discussed in greater details in the revised manuscript (page 10-11, lines 213-230, red text; page 26-28, lines 560-601, red text).*

*To further validate our results of the prediction by DENA, we were able to compare the DENA-identified m6A sites in Arabidopsis with data from a newly published database, REPIC[2], in which about 72% sites in our results were positively confirmed. We have included these new results in the revised manuscript (page 15, lines 313-316, red text). Thus, both results from our experimental validation and comparison with REPIC data indicates that DENA achieves reliable and consistent prediction of m6A modification sites.*

Reviewer #2:

This is an important paper addressing part of a problem in the epitranscriptomics field, the detection of modified nucleotides using direct RNA sequencing on the nanopore. The paper makes strong claims about the strength of the tool, but this is largely supported by their data.

DENA does appear to be a superior choice to many of the alternatives for calling modified sites in direct-RNA sequencing dataset. The software is accessible through a well-documented github (though, some proof reading is needed there!) and appropriately easy to use through python.

*Response: Thank you for the insightful comments and suggestions on our work. In the revised manuscript, we have addressed all the comments and performed many additional data analyses suggested by the reviewer.*

(1) The authors do a very important test of DENA against SCARLET, the current gold standard in site-specific m6A quantification, and the examples here do add credence to their claims that DENA outperforms other tools. Here however I think the manuscript needs more data to be shown, how does e.g xPore perform?

*Response: We appreciate the reviewer’s comment and affirmation of our work’s significance. We have summarized the existing comparison data and make the new efforts to show more data of comparison to additional programs like xPore[3], in order to satisfy the reviewer’s constructive suggestions.*

*In the last version of our manuscript, we compared the modification rates detected by DENA and Nanom6A[4] at the m6A sites identified by SCARLET in human gene transcripts. We found that DENA achieved the consistent modification rates with that of the SCARLET, while Nanom6A is consistent on some sites and showed tendency to overestimate the rates on some other sites (Figure 4j). Note we have added discussion about them in the revised manuscript (page 11-12, lines 237-253).*

*In the revision, we preformed new analysis to compare the predicted modification rates by DENA with those by other methods, including xPore, LEAD-m6A-seq[5] and Deoxyribozyme-based Method[6]. These new data are presented in a new Supplementary table in the revised manuscript (Supplementary information：Supplementary table 3). Please note that the modification rates predicted by DENA at seven sites, ACTB_5527743, BSG_583239, BSG_583346, TPT1_45337310, TPT1_45337303, YTHDF2_28743593 and PARP1_226361173, are in general agreement with those verified by at least one experimental method, SCARLET and/or LEAD-m6A-seq. Some details are dissected as follows:*

*For three m6A sites (ACTB_5527743, SEC11A_84669673 and MRPL20_1402080) identified by Deoxyribozyme-based Method, ACTB_5527743 and MRPL20_1402080 was affirmed by DENA, xPore and Nanom6A, while SEC11A_84669673 was confirmed by DENA and Nanom6A except xPore.*

*For five sites (ACTB_5527743, BSG_583239, YTHDF2_28743593, PARP1_226361173 and ACTG1_81511529) quantified by LEAD-m6A-seq, the modification rates predicted by DENA at the four sites (ACTB_5527743, BSG_583239, YTHDF2_28743593 and PARP1_226361173) were comparable with those quantified by LEAD-m6A-seq.*

*For SCARLET-quantified sites (ACTB_5527743, BSG_583239, BSG_583346, TPT1_45337310, TPT1_45337294 and TPT1_45337303), four sites (ACTB_5527743, BSG_583346, TPT1_45337310 and TPT1_45337303) were predicted by DENA with comparable modification rate. However, some overestimation of the rates were obtained by other models. For example, the modification rate of ACTB_5527743 quantified by SCARLET and LEAD-m6A-seq were 0.21 and 0.29, respectively (closed to 0.18 predicted by DENA), whereas 0.84 and 0.75 were predicted by xPore and Nanom6A.*

*One negative site (PARP1_226361173) that was designed as a control site by LEAD-m6A-seq in previous study[5], were consistently identified by DENA and xPore as unmodified-site.*

*Overall, the modification rates detected by DENA are generally consistent with those identified by experimental methods (SCARLET, LEAD-m6A-seq). Notably, the prediction of reduction in m6A modification rates in METTL3 knock-down mutant line using the three models, Nanom6A, xPore and DENA, were in general agreement. The manuscript has been updated with these new analysis (page 12-13, lines 253-267; red text; Additional file 1: Supplementary Table 3).*

(2) What m6A rates do other transcripts show in scarlett and other software? I wouldn't expect perfect performance from DENA in all scenarios (this would be remarkable) but I think this is a really important addition to allow the field to choose the best tool as needed.

*Response: We appreciate the reviewer’s comments.*

*For mRNA transcripts we have shown that DENA has a superb performance as in comparison with SCARLET, LEAD-m6A-seq, Nanom6A, xPore, etc. (Please refer to the discussion in the 1st question above).*

*Because the reference (Homo_sapiens.GRCh38.cdna.all.fa) from Ensembl does not contain non-coding RNA sequence, so we only identified the m6A sites for mRNAs (ACTB, TPT1 and BSG) in the last version of our manuscript, but not the two long non-coding RNAs (lncRNAs) (MALAT1 and TUG1) that were identified by SCARLET[7]. Per the reviewer’s comment, we again looked into those lncRNAs, MALAT1 and TUG1 by re-analysis. We found the Nanopore sequencing coverage of them were very low, both are fewer than 5 in all the datasets, which made it impossible to conduct a reliable analysis. We speculate about its cause and believe it is likely lncRNAs were not retained during the purification of mRNA using poly(T) before nanopore direct RNA-Sequencing, as the poly(A) structure may not be there for lncRNAs[8]. This is an important question for nanopore direct RNA-sequencing technology, which we have added discussion in the revised manuscript (page 11, line 231-237, red texts).*

*As discussed in above questions, DENA showed superb performance in comparison to Nanom6A and xPore on many of the m6A modification sites identified by experimental methods like SCARLET, LEAD-m6A-seq and Deoxyribozyme-based Method. Note for revision of our manuscript, we have also performed new experimental validation ourselves (please refer to question 5 of reviewer #1 for details). For selecting the best tool for analysis, we believe each software has a niche in certain circumstance, and would suggest to use a combination of multiple software when it is possible, and obtain the optimal results. However, based on the consistent performance DENA and its ease of use, we recommend to use DENA first.*

(3) The manuscript is clear and well written, it is however necessary to expand on how the DENA software works, considering the average reader of Genome Biology, rather than an expert reader in the machine learning field. The paper is a thorough exploration of how DENA performs, but only ~20 lines are dedicated to how it functions or how it was developed in the methods. This really does need to be expanded for the final publication.

*Response: We thank the reviewer for the comment. We have added description on the development of our model with more details for the revision of our manuscript. They have been added in the section of “Materials and methods” in the revised manuscript (page 22, lines 463-469; page 22, lines 476, 480-482; page 23, lines 485-496; page 24-25, lines 509-528; red text).*

*Reference*

*1. Xiao Y, Wang Y, Tang Q, Wei L, Zhang X, Jia G: An Elongation- and Ligation-Based qPCR Amplification Method for the Radiolabeling-Free Detection of Locus-Specific N(6) -Methyladenosine Modification. Angew Chem Int Ed Engl 2018, 57:15995-16000.*

*2. Liu S, Zhu A, He C, Chen M: REPIC: a database for exploring the N(6)-methyladenosine methylome. Genome Biol 2020, 21:100.*

*3. Pratanwanich PN, Yao F, Chen Y, Koh CWQ, Wan YK, Hendra C, Poon P, Goh YT, Yap PML, Chooi JY, et al: Identification of differential RNA modifications from nanopore direct RNA sequencing with xPore. Nature Biotechnology 2021.*

*4. Gao Y, Liu X, Wu B, Wang H, Xi F, Kohnen MV, Reddy ASN, Gu L: Quantitative profiling of N(6)-methyladenosine at single-base resolution in stem-differentiating xylem of Populus trichocarpa using Nanopore direct RNA sequencing. Genome Biol 2021, 22:22.*

*5. Wang Y, Zhang Z, Sepich-Poore C, Zhang L, Xiao Y, He C: LEAD-m(6) A-seq for Locus-Specific Detection of N(6) -Methyladenosine and Quantification of Differential Methylation. Angew Chem Int Ed Engl 2021, 60:873-880.*

*6. Bujnowska M, Zhang J, Dai Q, Heideman EM, Fei J: Deoxyribozyme-based method for absolute quantification of N (6)-methyladenosine fractions at specific sites of RNA. J Biol Chem 2020, 295:6992-7000.*

*7. Liu N, Parisien M, Dai Q, Zheng G, He C, Pan T: Probing N6-methyladenosine RNA modification status at single nucleotide resolution in mRNA and long noncoding RNA. RNA 2013, 19:1848-1856.*

*8. Yang L, Duff MO, Graveley BR, Carmichael GG, Chen LL: Genomewide characterization of non-polyadenylated RNAs. Genome Biol 2011, 12:R16.*

**Second round of review**

**Reviewer 1**

*The author almost addressed my concerns.*

*We carefully evaluated the final revised manuscript and response to Reviewer 2. The authors did lots of valuable work to address the reviewer 2 comments. They added a description of how the model constructed and worked. In addition, they also made a comparison with the DENA model and other models (like xPore, SCARLET). These results have confirmed the efficiency and accuracy of the DENA model.*

*There is also a key issue that gets our attention. Nanopore direct RNA-Sequencing used ploy(T) to purification mRNA may cause useful information lost, for example, as the authors explained that lncRNAs were not retained during the sequencing process. To some extent, computational work based on machine learning depends on the accuracy of the input experimental data. This would be better resolved in the future with the constant improvement of DRS sequencing technology.*

**Authors Response**

**Point-by-point responses to the reviewers’ comments:**

Reviewer #1:

The author almost addressed my concerns.

We carefully evaluated the final revised manuscript and response to Reviewer 2. The authors did lots of valuable work to address the reviewer 2 comments. They added a description of how the model constructed and worked. In addition, they also made a comparison with the DENA model and other models (like xPore, SCARLET). These results have confirmed the efficiency and accuracy of the DENA model.

There is also a key issue that gets our attention. Nanopore direct RNA-Sequencing used ploy(T) to purification mRNA may cause useful information lost, for example, as the authors explained that lncRNAs were not retained during the sequencing process. To some extent, computational work based on machine learning depends on the accuracy of the input experimental data. This would be better resolved in the future with the constant improvement of DRS sequencing technology.

*Response: According to the comment of Referee #1, we discussed the limits and the input experimental data of direct RNA sequencing in the detection of non-coding RNAs and their m6A modifications in the Conclusion section of the revised manuscript. Meanwhile, we give corresponding precautions or solutions to these limits, hoping to be helpful to researchers using direct RNA sequencing to detect RNA modification, especially on non-coding RNAs (Red words in the Conclusion section of the revised manuscript).*
